# The lockdown may contribute to the COVID-19 cases in developing countries, different perspectives on the curfew act, a report from Jordan

**DOI:** 10.1016/j.amsu.2020.12.018

**Published:** 2020-12-18

**Authors:** Nasr Alrabadi, Razan Haddad, Iyad Albustami, Ibrahim Al-Faouri, Nail Obeidat, Mohammad Al-Ghazo, Daher Al-rabadi, Adi Khassawneh

**Affiliations:** a Department of Pharmacology, Faculty of Medicine, Jordan University of Science and Technology, Irbid, 22110, Jordan; b Department of Pharmaceutical Technology, Faculty of Pharmacy, Jordan University of Science and Technology, Irbid, 22110, Jordan; c Faculty of Medicine, Jordan University of Science and Technology, Irbid, 22110, Jordan; d Department of Community and Mental Health Nursing, Faculty of Nursing, Jordan University of Science and Technology, Irbid, 22110, Jordan; e Department of Obstetrics and Gynecology, Faculty of Medicine, Jordan University of Science and Technology, Irbid, 22110, Jordan; f Department of Surgery and Urology, Faculty of Medicine, Jordan University of Science and Technology, Irbid, 22110, Jordan; g King Abdullah University Hospital, Jordan University of Science and Technology, Irbid, 22110, Jordan; h Division of Family Medicine, Department of Public Health, Faculty of Medicine, Jordan University of Science and Technology, Irbid, 22110, Jordan

**Keywords:** COVID-19, Pandemic, Curfew, Lockdown, Extended family, Developing countries

## Abstract

COVID -19 has driven an unprecedented challenge to the economic, social, and health aspects of human life worldwide. The daily increasing numbers of human life loss encourage us, the healthcare and public health communities, to share best practices and lessons learned to mitigate the resurgence of this pandemic. On the other hand, the pandemic itself or alternatively our policies in dealing with it has led to a dramatic loss and disastrous effects on many aspects including the food and nutritional systems and the world of work. The economic and social disruption caused by the pandemic is devastating; tens of millions of people are at risk of falling into extreme poverty, while the number of undernourished people, currently estimated at nearly 690 million, could increase by up to 132 million by the end of the year. The number of workers who are losing their jobs and the number of bankruptcies for small businesses are increasing. This report aims to bring the attention of policymakers, especially in the developing countries including Jordan, to different perspectives about crucial law acts, the lockdown and the curfew act, that have tremendous effects on the economy and may soon become a main contributor to the increased level of COVID-19 transmission and the main source of the new COVID-19 cases. We hypothesize for a mathematical model based on the comparison between the number of sporadic new cases, number of new cases/family, and the average number of family members to anticipate the value and the sufficiency of the lockdown or the curfew acts on modulating the transmission and the number of new COVID-19 cases in societies.

COVID -19 has driven an unprecedented challenge to the economic, social, and health aspects of human life worldwide. The daily increasing numbers of human life loss encourage us, the healthcare and public health communities, to share best practices and lessons learned to mitigate the resurgence of this pandemic. On the other hand, the pandemic itself or alternatively our policies in dealing with it has led to a dramatic loss and disastrous effects on many aspects including the food and nutritional systems and the world of work. The economic and social disruption caused by the pandemic is devastating; tens of millions of people are at risk of falling into extreme poverty, while the number of undernourished people, currently estimated at nearly 690 million, could increase by up to 132 million by the end of the year. The number of workers who are losing their jobs and the number of bankruptcies for small businesses are increasing. This report aims to bring the attention of policymakers, especially in the developing countries including Jordan, to different perspectives about crucial law acts, the lockdown and the curfew act, that have tremendous effects on the economy and may soon become a main contributor to the increased level of COVID-19 transmission and the main source of the new COVID-19 cases. We hypothesize for a mathematical model based on the comparison between the number of sporadic new cases, number of new cases/family, and the average number of family members to anticipate the value and the sufficiency of the lockdown or the curfew acts on modulating the transmission and the number of new COVID-19 cases in societies.

The social demographics of societies between east and west show major discrepancies in terms of household average, family shape, customs, and traditions. Of thus counting to different behaviors and responses to broad legislations. The risk of COVID-19 transmission increases with close contact, repetitive contact, and not wearing protective devices like masks. The demographics of societies in developing countries, including Jordan, show a tendency for having on average more than 4–5 members in every single family [1], as well, close or extended families are lean-to reside in extended patterns. From an observational point, social distancing and masking carry low-profile importance when an extended family assembles. Moreover, it is difficult to be applied in many societies or communities including some communities in Jordan and may have some noxious effects from social and psychological health aspects. On the other hand, international guidelines are not recommending routine social distancing and the use of protective devices within the same family [2].

We acknowledge that the family level of COVID-19 spreading is only a reflection of the infection running in work, shopping, and grocery places. Yet, obliging a weekly recurring curfew might facilitate the infection spreading by increase in-home contact and increase pre- and post-curfew transportation rush and shopping overcrowding. Thus, in return feed the vicious cycle of continuous infection from families to society and society to families. Many reports suggested a second surge of increasing the number of COVID-19 cases following the application of curfew [3]. It also suggested that the number of symptomatic (more severe) cases is higher than that of asymptomatic cases among this type of transmission.

Despite the well-known effect of social festivals, election campaigns, mass crowding in increasing the spreading rate and daily cases, extended families phenomena should also be considered a contributing factor for the infection rate and mass numbers of patients of the same family. More importantly, it may be considered a facilitator for spreading the infection among elderly and high-risk patients who reside at home and escaping away from the community, especially in the eastern societies where the elderly are taken care of by the families of their children. As well, patients with other diseases that are considered high-risk factors for death from COVID-19 are preferring to reside at homes to reduce the risk of infection from the community or the hospitals. Moreover, they are encouraged by policymakers and health care providers to stay at home as a safe strategy to reduce the risk of COVID-19 infection.

In Jordan; national reports indicated, 14 days after the mass gatherings for parliament election, that most of the provinces with known demographic of extended families have recorded the highest numbers of increment of cases numbers per 100,000 population (Irbid, Jarash, Tafileh, Karak, Mafraq, Balqa, Madaba, Aqaba, Maan, and Ajloun) (Table 1). While this increase, compared to the cumulative numbers before the elections, may be attributed to the crowding during the election, we also can assume that it is related to the 4-days curfew after the election especially with the pattern of increase in the provinces away from the capital with a high number of family members and where the effect of the extended family phenomena is higher. The increase in cases in society will be reflected on the family rates and case numbers, yet obligatory curfew and homestay regularly for one day weekly or few days after national events/crowding like the parliament elections, may accelerate the cross-contact point between healthy and infectious individuals within the same family. Comparing the sporadic numbers of COVID-19 cases to the numbers resides within the same family before and after the curfew can solidify such an argument. Currently, in Jordan, we are working toward getting those numbers and comparisons. Based on that, our strategies in dealing with the COVID-19 pandemic may differ.

**Table 1:** Daily cases/100k citizen/governorate: Comparing 14-days after the election (the day of submitting the manuscript) with the number before the elections. Updated daily data from the website of the Jordanian Ministry of health.

	Current daily cases/100k	Before 14 days	The 14-days change ratio
Ajloun	139.8	32.6	329.3%
Aqaba	207.3	69.0	200.6%
Jarash	118.0	42.8	175.8%
Al-Tafileh	103.7	43.6	137.8%
Al-Mafraq	38.8	16.8	130.4%
Al-Karak	78.4	38.1	105.7%
Madaba	65.4	31.8	105.4%
Irbid	46.0	25.7	78.8%
Al-Balqa	63.4	35.8	77.3%
Ma'an	83.9	66.0	27.2%
Amman	45.5	57.4	−20.8%
Zarqa	24.9	47.9	−47.9%

On the other hand, and from a hypothetical point of view, around 100,000–150,000 (for 3–4 weeks) confirmed new cases that are considered active cases were detected in Jordan. According to some reports, the total active cases which may not be detected can reach up to 20 times more than that [4,5]. Therefore, the active cases in Jordan may be close to 2 Million. This number represents 1:5 of the whole Jordanian population. Giving that the average family number is 4–5, then this number may indicate that there is a possibility of having an active case for each family. Therefore, the curfew and the lockdown may increase the transmission between family members more than the transmission within the society from one person to another. In agreement with that, some reports pointed to the low probability of society transmission when proper precautions are taken [6]. We understand that this calculation is hypothetical and may not be accurate given that the numbers of the cases are not distributed evenly between the families. But, from this hypothetical calculation, we are getting the impression that we became closer to this assumption and the lockdown or the curfew may contribute and will contribute in the future to the increase in the COVID-19 cases. We are trying to build such an accurate model for this scenario based on the updated numbers that we will receive from the center of crisis management in Jordan. This model can help us and other countries in the future to define when to apply or stop applying the curfew act.

Hopefully, this communication note will contribute to public health and policymakers discussions to actively prevent and control the transmission of COVID-19, to minimize the negative impact across developing and developed countries, to flatten the curve for the numbers of new cases, and to reduce the heavy load and pressure on health care systems. This is important at this stage because the percentage of the expected active cases in the society (1:5) may become close to the count of family members in Jordan (at least 5 per family) and other developing countries and at such a threshold holding the lockdown and the curfew laws may become critical. On the other hand, this will help in refreshing again the economic status and will help the society from many different economic and social aspects. However, we still encourage the prevention of some of the unnecessary social activities and events that have the potential to increase the transmission when high-intensity crowding is expected.

## Conflict of Interest Statement

The authors declared no conflict of interest

## Funding

Not Applicable.

## Provenance and peer review

Not commissioned, editor reviewed.
